# Real-time data fetching approach for performance evaluation of a DFIG wind power generation system using an IoT-enabled wind emulator

**DOI:** 10.1038/s41598-025-24234-x

**Published:** 2025-11-18

**Authors:** R. Sitharthan, M. Rajesh, R. Senthil Kumar, Shanmuga Sundar Dhanabalan

**Affiliations:** 1 Centre for Smart Grid Technologies, Vellore Institute of Technology, Chennai, Tamil Nadu India; 2 Department of Computer Science and Engineering, Aarupadai Veedu Institute of Technology, Vinayaka Mission’s Research Foundation (DU), Paiyanur, Chennai, Tamil Nadu India; 3 School of Electrical Engineering, Vellore Institute of Technology, Chennai, Tamil Nadu India; 4 School of Computing, Engineering and Mathematical Sciences, La Trobe University, Melbourne, VIC 3086 Australia

**Keywords:** Wind power generation system, Doubly-Fed induction generator, Wind emulator system, Performance evaluation, Internet of things, Cloud, FPGA, Energy science and technology, Engineering, Mathematics and computing

## Abstract

The increased integration of wind energy into the power system network requires advanced testing and performance evaluation methods to ensure reliability and efficiency. This paper presents an IoT-based real-time data collection method for analyzing the performance of the Wind Power Generation System (WPGS) using an intelligent IoT-enabled wind emulator system. The proposed system uses IoT to gather comprehensive real-time wind data, which is processed by a digitally controlled emulator to accurately assess wind turbine responses under different wind conditions. The advantage of this system is that IoT and cloud-based data analytics enable predictive analysis of the WPGS, helping evaluate its behavior and performance under various real-time scenarios. The approach is tested with a wind emulator setup consisting of a 1 kW Doubly-Fed Induction Generator (DFIG) connected to a Brushless DC (BLDC) motor, where the wind turbine model is developed on the VEE Pro platform, integrating an IoT-NodeRed, cloud API, and FPGA controller to simulate real-world wind conditions. Unlike conventional systems, the proposed architecture achieves real-time synchronization between global weather data and emulator control with low latency of 180 ms. Experimental results indicate 87% model accuracy Mean Absolute Percentage Error (MAPE) between theoretical and emulator power outputs, 95% health index reliability, and near-unity grid power factor of 0.999. This study provides a cost-effective, scalable, and adaptable solution for real-time wind energy analysis, supporting ongoing research and development.

## Introduction

In recent years, there has been a noticeable worldwide increase in energy demand. Considering the rising energy needs, emissions, sustainable development, and the exhaustion of fossil fuels, the energy sector has increasingly shifted toward Renewable Energy Resources (RER) for power generation. Among various RER, wind energy plays a key role in energy supply and is highly regarded for its technological advancements, low maintenance, and longevity^1^. Currently, wind energy makes up about 8–10% of global electricity generation^2^. Notably, countries such as Denmark, Germany, and the United Kingdom have achieved higher shares, with around 35–50% of their electricity produced through Wind Power Generation Systems (WPGS)^3^. Therefore, WPGS are essential for energy contribution, and large-scale integration of WPGS into existing power grids is crucial to meet the growing energy demands^4^.

Among different WPGS technologies, the Doubly-Fed Induction Generator (DFIG)-based system is notable for its ability to operate at variable speeds and reduce power fluctuations, making it a preferred choice for grid-connected applications^5^. However, the unpredictable and intermittent nature of wind poses significant challenges to grid stability. During grid disturbances, the direct connection of the DFIG stator to the grid can cause voltage dips and surges in current, which may lead to excessive stress on the rotor circuit, increased DC-link voltage, and potentially severe mechanical and electrical damage^6^. Such faults not only risk disconnecting WPGS from the grid but also threaten overall system stability. Therefore, studying the behavior of DFIG-based WPGS under various wind conditions and environmental factors is essential before installation.

Several studies employing different methods emphasize the importance of investigating WPGS performance with a wind emulator system. In^7^, a flexible control platform built with a dSPACE prototype was developed for an 11-kW wind energy system operating in both grid-connected and standalone modes. This setup enabled quick implementation and testing of complex control strategies, including MPPT and back-to-back converter control using Simulink. In^8^, a wind turbine simulator was created using an inverter-driven induction motor to imitate real wind turbine shaft torque for testing wind energy conversion systems. Controlled via C-based software and real-time interfaces, the simulator demonstrated accurate and practical performance for evaluating drive trains, converters, and controllers. In^9^, a DC machine-based emulator was developed to simulate the mechanical drive train of a DFIG-based wind system using a three-mass oscillation model. This system allows studying the DFIG’s characteristics under various scenarios. In^10^, a real-time wind turbine emulator employing sliding mode control has been proposed. The system replicates a wind turbine, supporting the study of static and dynamic turbine characteristics through a DC motor-based setup. The control strategy developed has improved performance, and the comparative study shows enhancements over traditional systems. However, the system’s comparison of traditional controllers’ performance only considers response time, overshoot, and accuracy. Additional grid analysis is necessary to fully assess the system’s overall performance.

The roles of IoT and AI have gained increasing importance in recent years. Their use in a real-time WPGS emulator study has significantly enhanced system intelligence, efficiency, and reliability^11^. In^12^, an IoT-enabled wind emulation platform was developed to improve the traceability and efficiency of wind energy systems using both real-time and forecasted wind data. Modeled in VEE Pro and integrated with FPGA and cloud APIs, the system allows real-time performance analysis and secure communication through ESP8266. In^13^, a comprehensive review of IoT simulators was presented, focusing on their capabilities and identifying key gaps related to scalability, energy models, and renewable energy integration. The study emphasizes the need for a flexible simulator that addresses IoT-specific challenges like security threats and VANET integration. Computational modeling and simulations are crucial tools for analyzing and optimizing wireless communication systems, offering insights into network behavior, protocol performance, and system design. Despite challenges with model accuracy and computational demands, they significantly enhance reliability, efficiency, and innovation in wireless network development^14–16^. These methods improve design efficiency and dependability despite issues related to model precision and computational resource requirements. In^17^, a Hybrid Controller (HC) combining ANFIS and PI controllers improves wind turbine emulator accuracy by reducing overshoot and settling time. Other studies demonstrate that sliding mode control and IoT integration enhance dynamic response and real-time wind condition simulation. The comprehensive review^18^ analyzed over 140 studies on Wind Turbine Emulators (WTE), highlighting the benefits of lab-scale WTEs, such as controlled conditions and space efficiency. The review details the prime movers, generators, and control strategies used, discussing their advantages and disadvantages to help researchers choose suitable WTE methods. In^19^, a study presents a low-power, lab-scale wind turbine emulator that accurately replicates real turbine behavior, providing a safe and controllable platform for research and education. Most existing research focuses on improving control accuracy for wind turbine emulators but often neglects issues like system scalability and real-world environmental variability. Additionally, IoT-integrated solutions face challenges in ensuring robust security and seamless real-time data processing.

To tackle these challenges, this paper presents an innovative real-time performance analysis framework for WPGS, using an intelligent Internet of Things (IoT)-based wind emulation system. Comparison of the proposed work with recent IoT-enabled wind emulator systems is listed in the Table 1. The proposed setup collects real-time global wind data via an IoT-API from multiple locations and reproduces corresponding wind profiles through a digitally controlled emulator. This system enables precise testing of wind turbine responses under various operating conditions, offering valuable insights into performance, grid compatibility, and fault tolerance. The study is conducted on an experimental platform consisting of a 1 kW DFIG driven by a Brushless DC (BLDC) motor, which is simulated and managed using VEE Pro software integrated with an FPGA controller and IoT cloud API. The results verify the emulator’s accuracy in replicating real-world wind conditions and demonstrate its potential to improve predictive maintenance, optimize grid integration, and support advanced wind energy research and development.

**Table 1:** Comparison of the proposed work with recent IoT-enabled wind emulator systems.

Reference	Platform and control	IoT/cloud integration	Predictive analytics	Emulator power	Key limitation	Novelty of proposed work
^12^	LabVIEW + ESP8266	Node-RED + Cloud	No	500 W	No predictive layer	Real-time global data + FPGA integration
^18^	Microcontroller	Limited	No	1 kW	No cloud analytics	Cloud–FPGA fusion with predictive HI
^20^	SCADA-based hybrid	Yes	Partial	3 kW	High latency	Secure IoT protocol + low-latency streaming
Proposed Work	VEE Pro + FPGA and ESP8266	Full MQTT/HTTPS + ThingSpeak Cloud	Yes (HI-based)	1 kW	Modular, scalable	Predictive, low-cost, real-time global emulator

The paper is organized as follows: Section “IoT-wind emulation system” explains the architecture of the IoT-based wind emulation system. Section “Modelling of wind emulation system” details the modeling of the wind emulation system, including the wind turbine model, chopper drive model, and DFIG model. Section “IoT cloud API-based data fetching for wind emulation system” covers the IoT Cloud API-based data collection for the wind emulation system, including wind data retrieval via the API and sensors, the user interface, and security concerns. Section “Experimental setup and implementation details” describes the setup of the experimental system. Section “Results and discussion” presents the results obtained and their validation. Section “Conclusion” provides a summary of the research findings and conclusions.

## IoT-wind emulation system

**Fig. 1:**
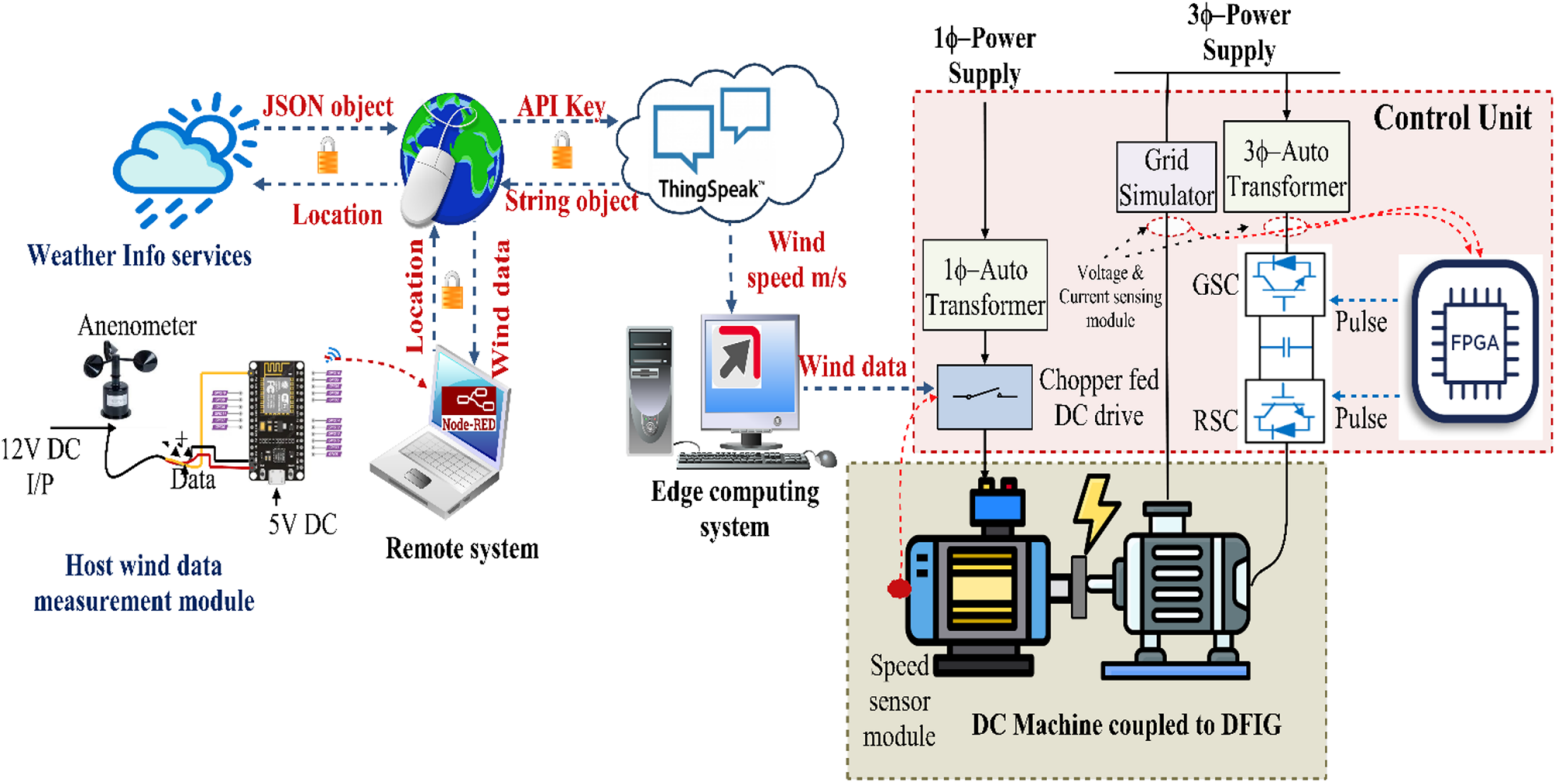
IoT-enabled wind emulation system.

Figure 1 shows the comprehensive IoT-enabled system for real-time wind data collection and wind turbine emulation using a Doubly-Fed Induction Generator (DFIG). Initially, the system retrieves wind data from a cloud-based weather information provider globally, while an anemometer setup installed at the host location measures local wind speed. The global wind data is processed by a remote system equipped with Node-RED, a flow-based IoT data management platform. Additionally, the locally measured wind speed is processed by an ESP8266 microcontroller and transmitted to a system running Node-RED^20^. The remote system connects to the cloud platform ThingSpeak via APIs, sending wind data along with JSON objects and API keys. ThingSpeak stores and visualizes this data, making it accessible to the edge computing system.

On the other hand, ThingSpeak stores and visualizes data from open weather info services through the API in the edge computing system. The edge system retrieves wind data from ThingSpeak in CSV files and feeds it into the DC drive control system to simulate real-time wind speed conditions. This system uses a chopper-fed DC drive to control the mechanical input to the BLDC, mimicking the mechanical output of a wind turbine. The DC machine is directly coupled to a DFIG, which operates with power electronics, including a Grid Side Converter (GSC) and a Rotor Side Converter (RSC) controlled by an FPGA-based control unit. This setup replicates real wind conditions for performance evaluation, grid integration studies, and control algorithm validation of wind energy systems.

## Modelling of wind emulation system

The developed wind emulation system includes a DFIG coupled with a DC machine and functions as the Wind Power Generation System (WPGS). The DC machine is powered via a chopper drive, which acts like an aerodynamic turbine. The DFIG’s stator is directly connected to the grid, while its rotor connects through a back-to-back Voltage Source Converter (VSC) consisting of a Grid Side Converter (GSC) and a Rotor Side Converter (RSC)^6^. The GSC manages DC-link voltage and reactive power, while the RSC controls speed and torque for optimal power output. The system transforms wind power into mechanical energy and subsequently into electrical energy.

### Wind turbine modelling

The power generated in a wind turbine by aerodynamics is defined as *P*_*m*_ and is expressed as,

1 $$P_{m}=0.5\rho C_{p}(\lambda,\beta)AW^{3}$$

**Fig. 2:**
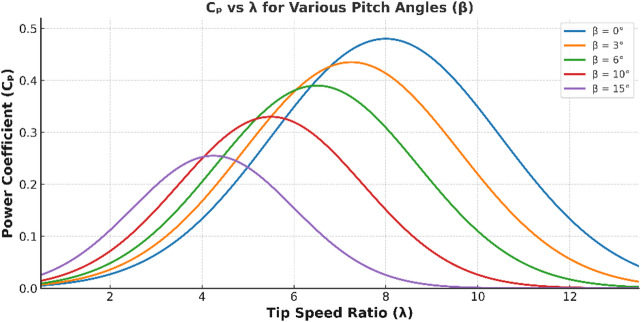
Wind turbine power coefficient versus tip speed for various pitch angle.

where *P*_*m*_ is characterized by *C*_*p*_ (*λ*,* β*), a dimensionless wind-turbine power coefficient determined by the manufacturer to maximize power capture. *C*_*p*_ *(λ*,* β)* depends on the tip speed ratio *λ* and pitch angle *β*. $\rho$represents air density (typically 1.225 kg/m³), *A* is the rotor swept area (m^2^), and *W* is wind speed (m/s)^21,22^. Figure 2 shows the power coefficient characteristic curve of the wind turbine. The *C*_*p*_ *(λ*,* β)* is expressed as,

2 $$C_{p}(\lambda,\beta)=0.52\left({\frac{{116}}{{\lambda_{j}}}-0.4\beta-5}\right)e^{{-\frac{{21}}{{\lambda_{j}}}}}+0.0066\lambda$$

3 $$\lambda=\frac{{\omega_{r}^{*}R}}{W}$$

from Eq. (3), (*ω*_*r*_) is the rotor rotational speed and *R* is the swept area radius of the turbine blades, respectively.

4 $$T_{m}=\frac{{P_{m}}}{{\omega_{r}}}$$

The mechanical torque (*T*_*m*_) is derived from Eq. (4) using mechanical power (*P*_*m*_) and optimal rotor speed *ω*_*ropt*_. This calculated *T*_*m*_ is then fed into the DFIG generator via a gear shaft system.

### Modelling of the chopper-fed DC motor

**Fig. 3:**
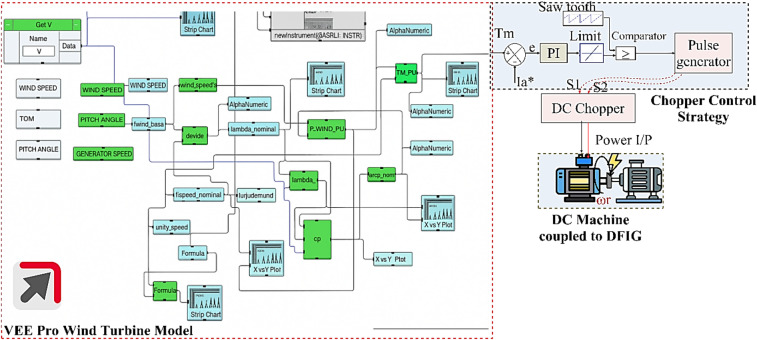
Chopper-fed DC motor control strategy.

A single-quadrant chopper controls the average voltage supplied to a DC motor by rapidly switching the supply on and off, regulating the motor’s speed. Figure 3 illustrates the chopper-fed DC motor control strategy. The system uses real-time wind speed in m/s to regulate the motor’s speed by adjusting the supply voltage. The electrical differential equation of the DC motor is given as,

5 $${V_a}(t)={R_a}{i_a}(t)+{L_a}\frac{{d{i_a}(t)}}{{dt}}+{K_e}\omega (t)$$

where, *V*_*a*_ and *i*_*a*_ are the armature voltage (*V*) and current (*A*), *R*_*a*_ and *L*_*a*_ are the armature resistance (*Ω*) and inductance (*H*), Further, *e*_*b*_*(t)* is the back emf (*V*) and *K*_*e*_ is the back emf constant. The angular speed (rad/s) is given by *ω(t)*. The DC motor mechanical differential equation is given as,

6 $$J\frac{{d\omega (t)}}{{dt}}+B\omega (t)={K_t}{i_a}(t) - {T_L}(t)$$

where, *T*_*m*_ is the electromagnetic torque (Nm), (i.e.,) *T*_*m*_ = *K*_*t*_ *. i*_*a*_*(t)*, *J* is the moment of inertia (kgm^2^). Further, *B* is the viscous friction coefficient (N ms) and *T*_*L*_*(t)* load torque (Nm). The average chopper model is represented mathematically as,

7 $${V_a}=\alpha {V_s}$$

where, α is the duty cycle (i.e.,) (0 < α < 1) and V_s_ is the DC input supply voltage. The complete state-space model of the chopper fed DC machine is given as,

8 $$\frac{{d{x_1}}}{{dt}}=\frac{1}{{{L_a}}}(\alpha {V_s} - {R_a}{x_1} - {K_e}{x_2})$$

9 $$\frac{{d{x_2}}}{{dt}}=\frac{1}{J}({K_t}{x_1} - B{x_2} - {T_L})$$

where, the state variables of the system are assumed to be *x*_1_ = *i*_*a*_*(t)* and *x*_2_ *= ω(t).*

### Modelling of DFIG

In a DFIG system, the RSC and GSC are usually modeled using voltage-space vector equations in the synchronous reference frame (d–q frame). Below are the general dynamic equations for both RSC and GSC control. The RSC manages rotor currents for active and reactive power control. The general dynamic equations in the d–q frame are provided as^4^.

10 $${v_{dr}}={R_r}{i_{dr}}+\frac{{d{\psi _{dr}}}}{{dt}} - {\omega _s}{\psi _{qr}}$$

11 $${v_{qr}}={R_r}{i_{qr}}+\frac{{d{\psi _{qr}}}}{{dt}}+{\omega _s}{\psi _{dr}}$$

where, *v*_*dr*_ and *v*_*qr*_ are the d–q axis rotor voltages, *i*_*dr*_ and *i*_*qr*_ are the d–q axis rotor currents, *ψ*_*dr*_ and *ψ*_*qr*_ are the d–q axis rotor flux linkages, *R*_*r*_ is the rotor resistance, *ω*_*s*_ is the synchronous speed. The flux linkages of the DFIG are given as,

12 $${\psi _{dr}}={L_r}{i_{dr}}+{L_m}{i_{ds}}$$

13 $${\psi _{qr}}={L_r}{i_{qr}}+{L_m}{i_{qs}}$$

where, *L*_*r*_ is the rotor inductance, *L*_*m*_ is the mutual inductance, *i*_*ds*_ and *i*_*qs*_ are the stator d–q currents (assumed known or constant). The GSC controls DC-link voltage and manages reactive power exchange with the grid. The generalized grid side voltage equations in d–q frame are given as,

14 $${v_{dg}}={R_f}{i_{dg}}+{L_f}\frac{{d{i_{dg}}}}{{dt}} - {\omega _s}{L_f}{i_{qg}}+{v_{d\_grid}}$$

15 $${v_{qg}}={R_f}{i_{qg}}+{L_f}\frac{{d{i_{qg}}}}{{dt}} - {\omega _s}{L_f}{i_{dg}}+{v_{q\_grid}}$$

where, v_dg_ and v_qg_ are the d–q voltages from GSC, *i*_*dg*_ and *i*_*qg*_ are the d-q currents injected by GSC, *R*_*f*_, and *L*_*f*_ are the filter resistance and inductance, respectively. Moreover, *v*_*d_grid*_ and *v*_*q_grid*_ are the d–q grid voltages (often *v*_*q_grid*_ = 0 in grid-aligned frame). The real and reactive power generated by DFIG is given as,

16 $${P_s}=\frac{3}{2}({v_{ds}}{i_{ds}}+{v_{qs}}{i_{qs}})$$

17 $${Q_s}=\frac{3}{2}({v_{qs}}{i_{ds}} - {v_{ds}}{i_{qs}})$$

where *Pe* and *Q*_*e*_ are the real and reactive power fed to the grid, respectively.

## IoT cloud API-based data fetching for wind emulation system

**Fig. 4:**
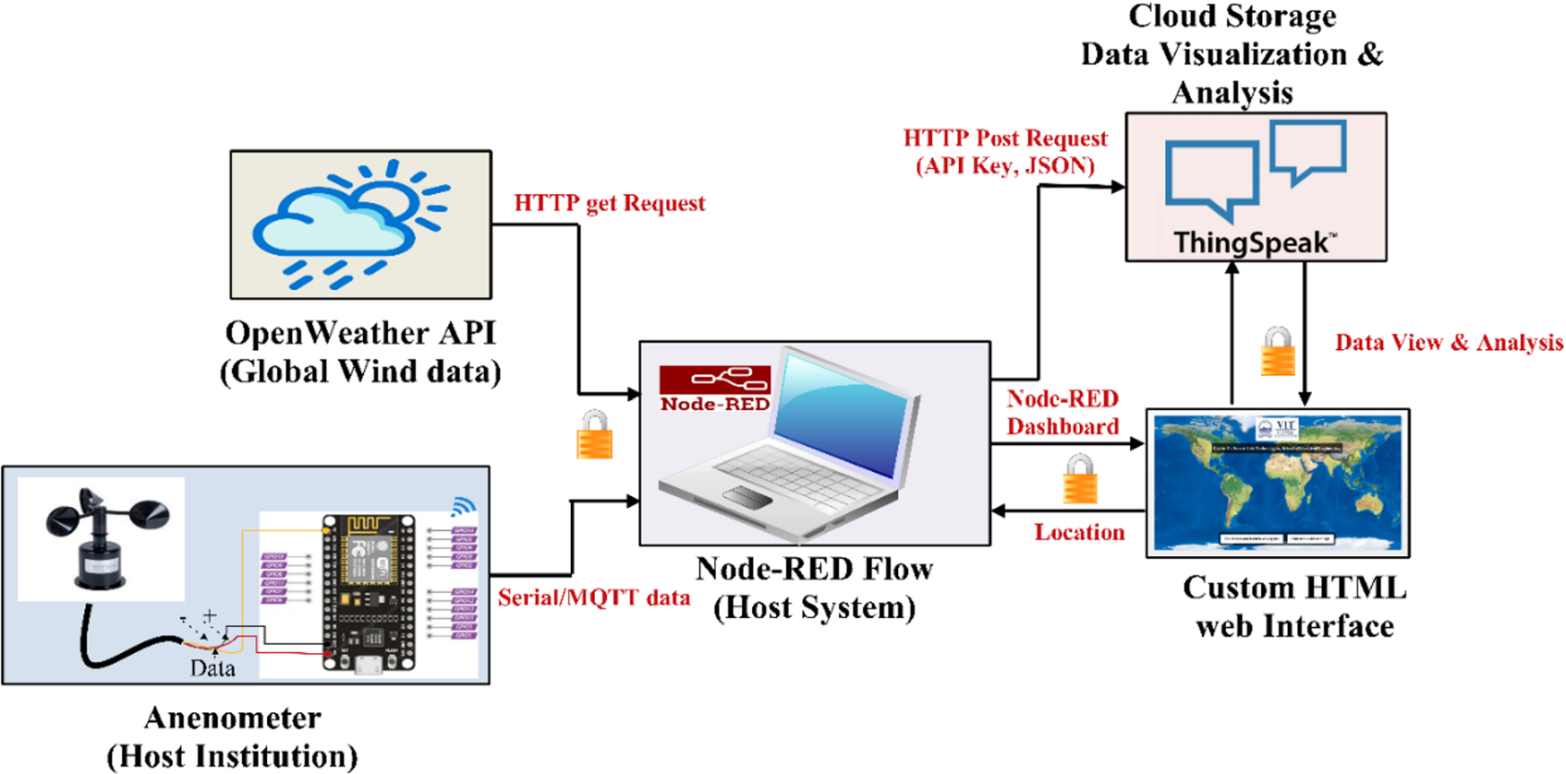
Real-time IoT cloud API-based data fetching.

The work incorporates Node-RED, a flow-based IoT tool, to collect and process global wind data from the OpenWeather API, as shown in Fig. 4. The custom HTML web page communicates with both Node-RED and ThingSpeak. A Node-RED dashboard, linked in the HTML page, enables users to input latitude and longitude to retrieve wind data from OpenWeather^23^. The OpenWeather site supplies real-time wind speed data based on specific geographical coordinates provided by the user.

Meanwhile, an anemometer is installed at the host institution to measure local wind conditions. Both data streams (global and local) are sent to ThingSpeak via Node-RED using the MQTT protocol. The data are stored on the ThingSpeak-MATLAB cloud server through the IoT platform, and the real-time data is displayed on the ThingSpeak dashboard. Additionally, this data can be used for further analysis with artificial intelligence^24^.

### **IoT wind data fetching unit**

**Fig. 5:**
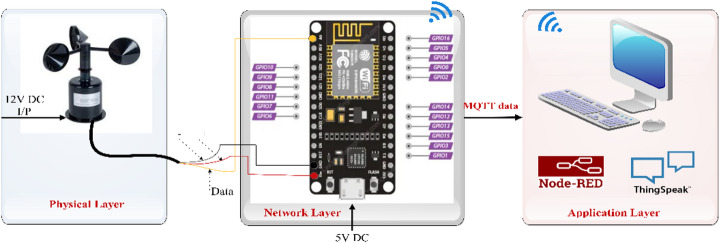
IoT-based wind measurement.

The IoT-based wind measurement system architecture, as shown in Fig. 5, combines physical, network, and application layers to measure, transmit, and display wind speed data. The physical layer includes an anemometer powered by a 12 V DC input, which captures real-time wind speed data and sends a pulsed signal to the network layer. In the network layer, a ESP8266 Wi-Fi module, powered by a 5 V DC input, processes the sensor’s signal through GPIO pins. This module transmits wind data via MQTT over Wi-Fi to the application layer. The application layer consists of Node-RED, which manages data processing and flow control, and ThingSpeak, which stores and visualizes the data in graphical form. The entire system operates smoothly to support IoT-based real-time wind monitoring and visualization.

### User interface and API architecture development

**Fig. 6:**
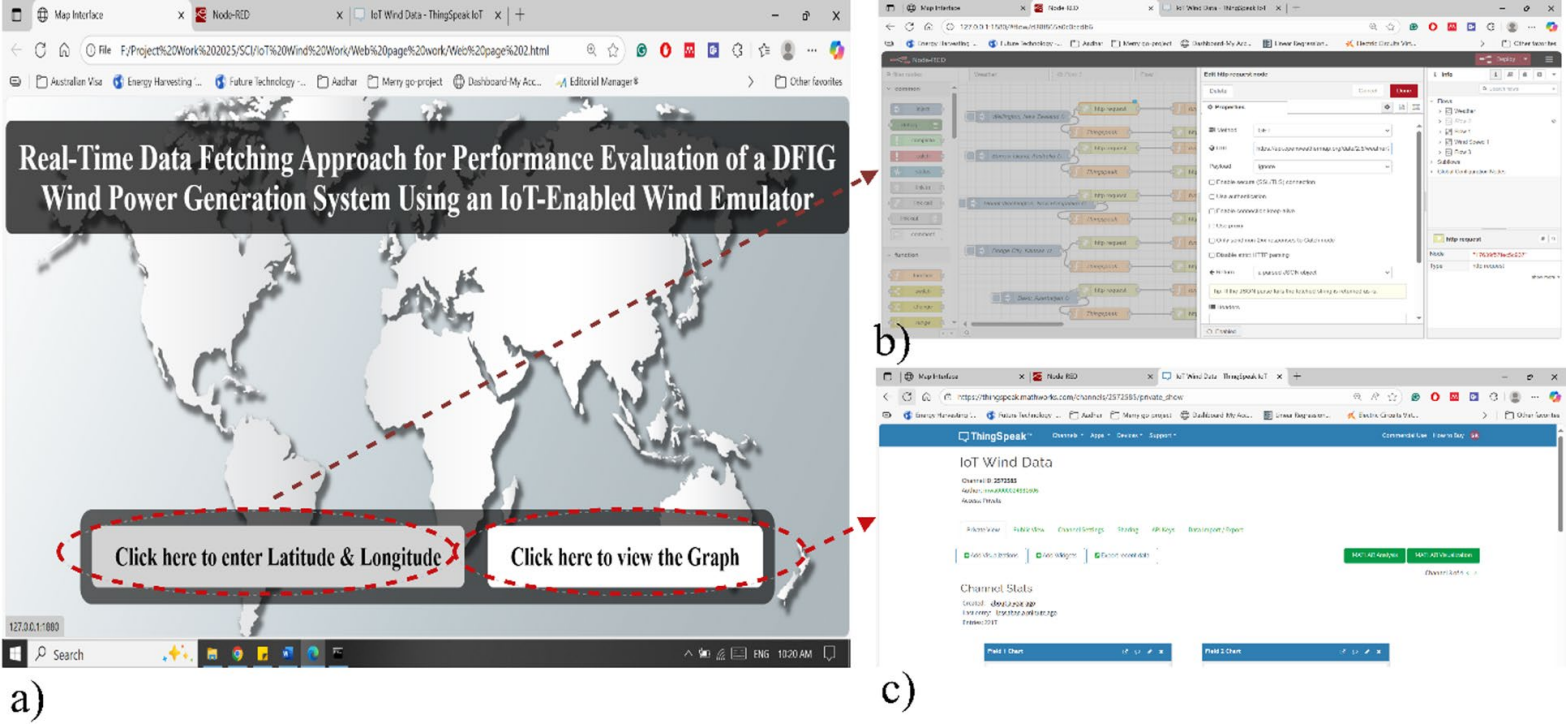
User interface, (**a**) HTML web interface, (**b**) Node-Red Platform, (**c**) Thinkspeak dashboard.

**Fig. 7:**
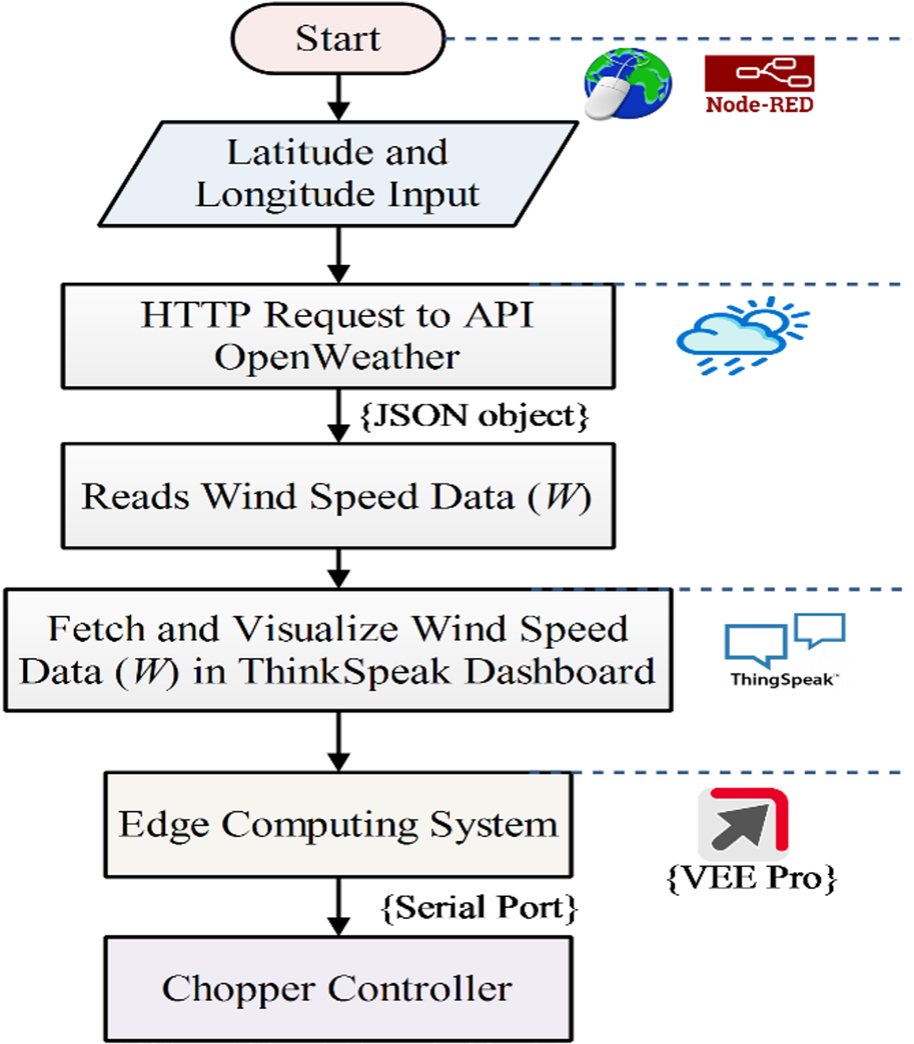
Fetching global wind data and data communication with the controller.

To facilitate user interaction, a custom HTML web page has been created, as shown in Fig. 6a. The webpage provides direct access to two key services: (i) the Node-RED dashboard for real-time input of latitude and longitude, and (ii) the ThingSpeak visualization page displaying wind data graphs, as shown in Fig. 6b and c. This HTML interface connects external web services, offering a streamlined experience for data input and visualization. Figure 7 illustrates the Node-RED flow API architecture system that fetches global wind data and manages data communication with the controller. The Node-RED flow API system integrates multiple components to establish a robust and efficient data flow. Node-RED acts as the central hub, receiving global wind data from OpenWeather via RESTful API GET requests and simultaneously incorporating local wind data from an anemometer through serial protocols. The combined dataset is then sent to ThingSpeak using HTTP POST requests, employing channel API keys and structured JSON objects for seamless integration. ThingSpeak functions as a cloud platform for data storage and visualization, displaying the data in interactive graph formats. Furthermore, using the serial port, wind data is supplied as input to the copper controller via VEE Pro.

To ensure security, ThingSpeak API keys are utilised for authentication and authorisation, preventing unauthorised data uploads and access. All data exchanges with ThingSpeak and OpenWeather occur over HTTPS to guarantee secure transmission and safeguard against eavesdropping. The Node-RED system hosted locally (on http://127.0.0.1:1880/) minimises exposure to external threats. Additionally, Node-RED employs error-handling flows for data validation, ensuring integrity before transmitting data to ThingSpeak. The wind data channels on ThingSpeak are configured as private, accessible only with valid credentials, further enhancing the system’s data integrity and security.

### Security protocol for IoT wind data fetching system

The IoT wind data fetching system uses a strong security protocol with multiple protection layers. For data authentication and authorization, ThingSpeak API keys are employed to ensure secure data uploads and access—private write keys are for uploads, and read keys are for viewing data. Likewise, OpenWeather API authentication is enforced through secure API key use, ideally restricted to specific IP ranges when supported, with all requests sent via HTTPS to prevent eavesdropping. The Node-RED dashboard is secured through username/password authentication, and additional security measures such as OAuth2 or JWT tokens can be added to control access.

For data integrity and security, HTTPS is required for all external API communications, including OpenWeather and ThingSpeak, to protect data during transmission. When working with local data via MQTT, MQTTS (MQTT over TLS) is recommended. Node-RED checks incoming data formats and values (e.g., wind speed ranges and units) to ensure only accurate data is sent to ThingSpeak^25–29^. For monitoring and incident response, logging should be enabled across Node-RED and ThingSpeak, with real-time alerts for suspicious activities such as multiple failed login attempts or API rate-limit breaches. Regular backups of Node-RED flows, configuration files, and ThingSpeak data channels are essential, along with a disaster recovery plan for quick restoration if the system is compromised. Physical security measures should be implemented to protect the anemometer and Node-RED hardware from tampering, and power backup systems should be used to maintain operation during outages.

### Communication performance and latency evaluation

The IoT–Cloud data transfer was analyzed for latency and reliability using Node-RED MQTT logs and ThingSpeak acknowledgment timestamps. The measured average end-to-end latency between data request and emulator update was 180 ms, with a maximum of 265 ms under peak API traffic. Message delivery success exceeded 98.3%, ensuring reliable emulator response. Table 2 summarizes the communication performance parameters.

**Table 2:** IoT communication and data reliability metrics.

Parameter	Average value	Remarks
End-to-end latency	180 ms	MQTT + HTTPS combined
Data delivery reliability	98.3%	Confirmed by ThingSpeak logs
Data packet loss	1.7%	Mainly due to Wi-Fi fluctuation
Average payload size	512 Bytes	JSON object per wind data update
Security protocols	HTTPS, MQTTS, API key	Multi-layer protection

## Experimental setup and implementation details

**Fig. 8:**
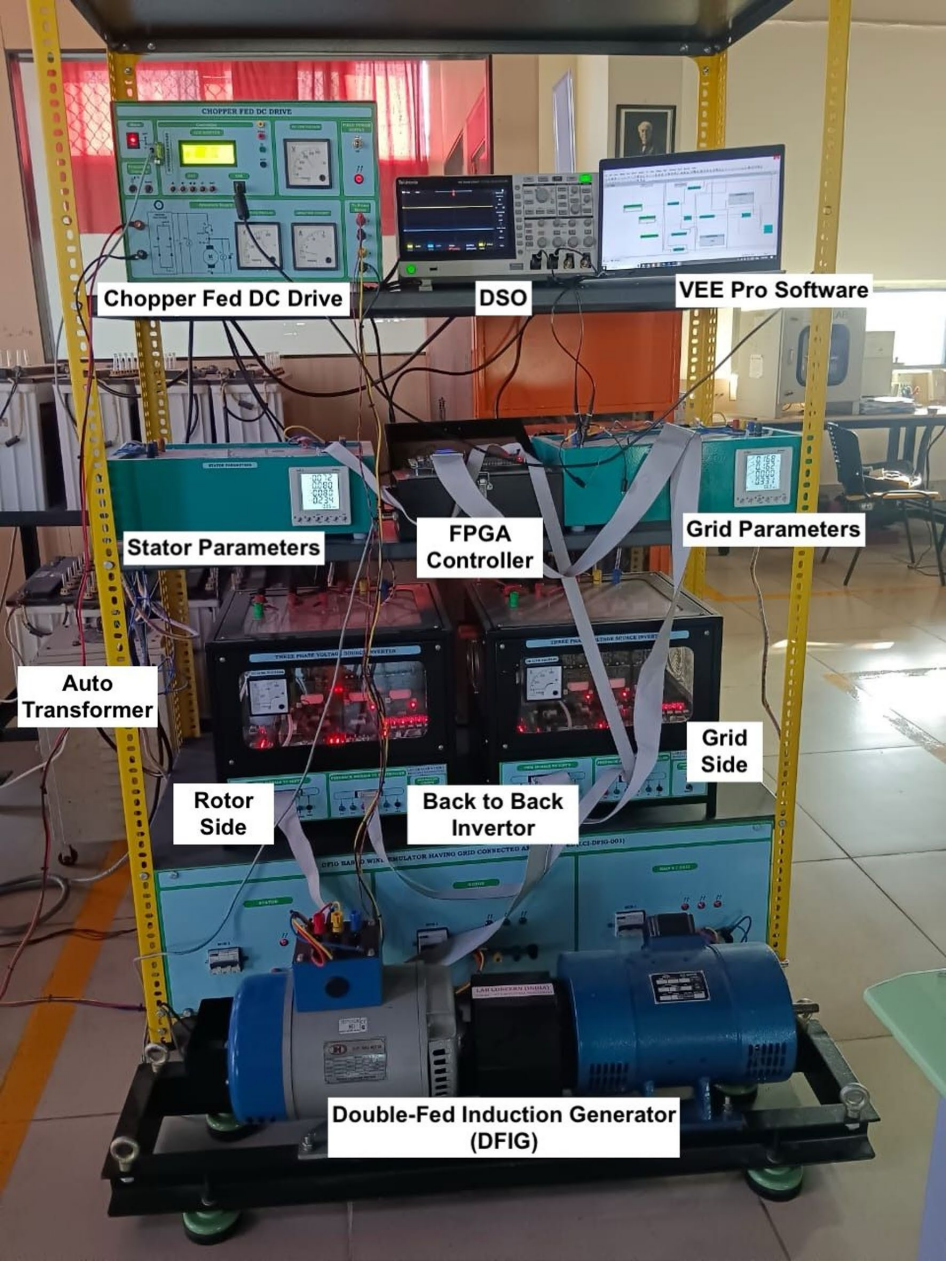
Studied IoT-enabled DFIG-based wind emulation experimental setup.

The experimental setup of the proposed DFIG-based wind emulation system is shown in Fig. 8. It consists of a modular, laboratory-scale system that mimics the behavior of a real-time wind power generation system using hardware components, advanced sensing modules, and an FPGA-based control architecture. The setup’s core features a chopper-fed DC motor drive acting as the main mover, mechanically connected to a three-phase, 1.5 HP AC slip ring induction generator. The rotor of the slip ring machine interfaces with a back-to-back Voltage Source Inverter (VSI) made up of two 1 kVA, 230 V (per phase), 10 A inverters with IGBT switches (model: SKM 100GB12T4), controlled through Semikron driver circuits. The inverters are supported by DC link capacitors rated at 2200 µF and 450 V, and the system operates at a DC link voltage of 350–380 V. The full specifications of the IoT-enabled DFIG-based wind emulation system are provided in Table 3.

**Table 3:** Specifications of DFIG-based wind emulation system.

Component	Specification
Rotor-side inverter (VSI)	1 kVA, 230 V/phase, 10 A, 6 IGBTs (SKM 100GB12T4)
Grid-side inverter (VSI)	1 kVA, 230 V/phase, 10 A, 6 IGBTs (SKM 100GB12T4)
DC link capacitors	2 × 2200 µF, 450 V
DC link voltage	Nominal: 350 V, Max: 380 V
Slip ring induction motor	1.5 HP, 415 V, 2.4 A, 1500 RPM
DC motor	1 HP, 220 V, 4.5 A, 1500 RPM
FPGA controller	Artix-7 200T
Driver circuits	Semikron
Multifunction meter	MFM 384 C, Rated 5 A (with CT 30 A/5A)
Voltage/current sensing modules	2 sets (Stator & Grid)
Autotransformers	1 three-phase and 1 single-phase
Chopper-fed DC drive	Connected to a DC motor
Software	VEE Pro with VISA Interface
IoT interface	ESP8266, Node-RED, ThingSpeak

To accurately monitor input and output electrical parameters, the system integrates Multifunction Energy Meters (MFM)-384 C, capable of measuring voltage, current, active and reactive power, frequency, and power factor. These MFMs are connected through current transformers (30 A/5A) to accommodate higher current ratings. The rotor and stator machine terminals are connected to three-phase autotransformers for grid simulation and adjustable voltage injection. Additionally, the three-phase VSI on the rotor side controls the DFIG’s rotor excitation. Meanwhile, the grid-side VSI ensures synchronization and regulated power delivery into the simulated grid through passive inductors.

An FPGA controller (Artix-7 200T) functions as the real-time control unit, generating switching pulses for both the RSC and GSC based on real-time feedback and control algorithms. The FPGA receives voltage and current data via sensing modules and responds to parameter variations and control logic defined in the system software. The controller keys (PSW2, PSW3, SW1–SW4) enable manual control of DFIG and grid pulse activation, as well as fine-tuning of DC link voltage and output power levels. The VEE Pro software environment is used to program and implement real-time control algorithms, interfacing with the FPGA through standard communication protocols such as VISA, which supports serial, GPIB, and VXI-based communication with measurement hardware. This setup allows flexible deployment, real-time monitoring, and efficient data handling. Furthermore, the modular FPGA–IoT hardware configuration offers substantial cost advantages compared to conventional SCADA or LabVIEW-based setups. The total hardware cost including FPGA controller, ESP8266, chopper drive, and sensor is approximately INR 10,00,000.

### Implementation process

**Fig. 9:**
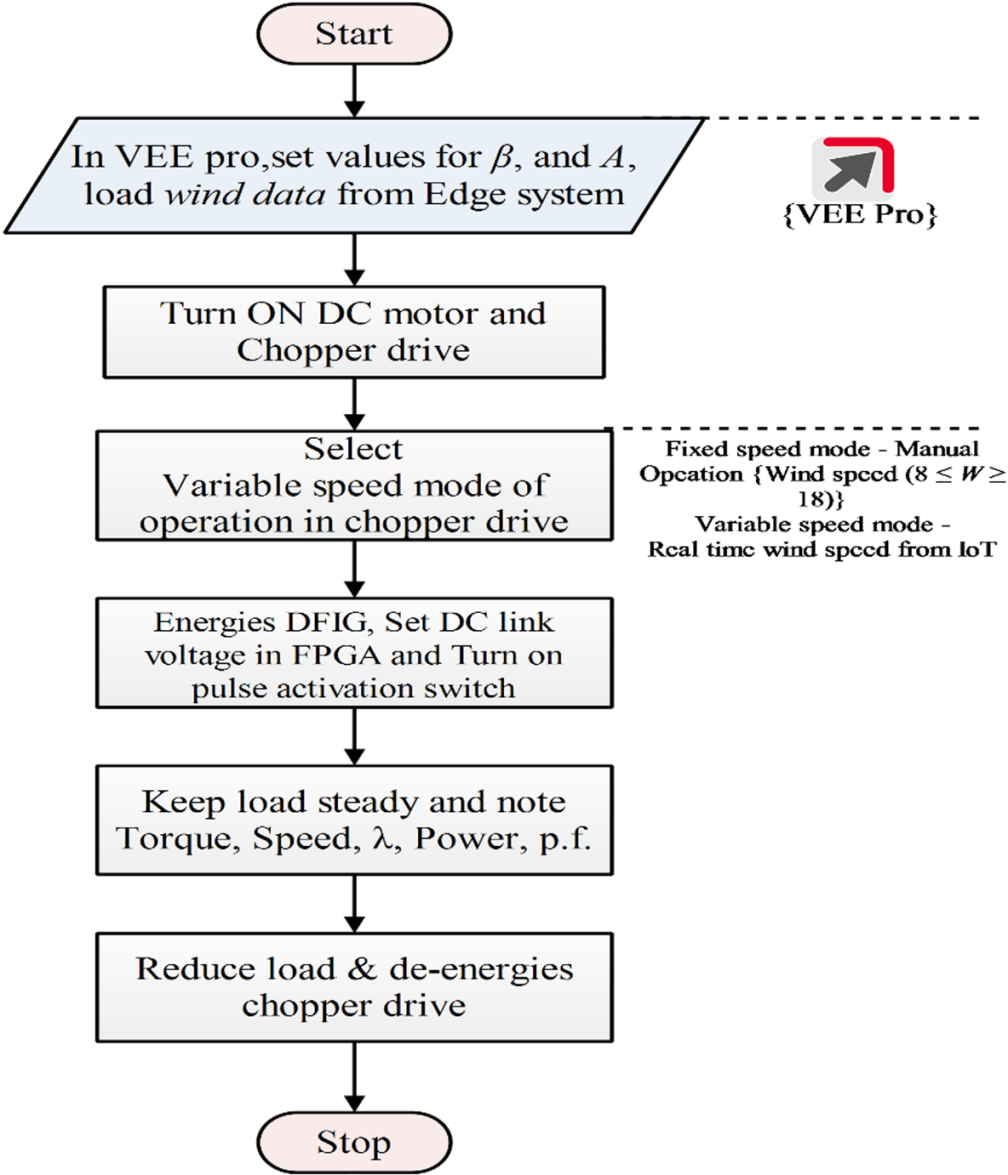
Implementation process.

The wind emulation unit is designed to evaluate the performance of a wind energy generation system using its characteristic curves. In the studied experimental setup, there are two operating modes: (i) Fixed Wind Mode—The wind energy conversion system is examined by varying system parameters at a constant wind velocity, with values set between 8 and 18 (8 ≤ W ≥ 18). (ii) Variable Wind Mode—The system is analyzed by changing wind velocity using data fetched from IoT via VEE Pro, while keeping the design parameters constant. Figure 9 shows the control process. The process begins in VEE Pro, where the pitch angle (β) and swept area (A) are set, and wind data is loaded from the edge system. The DC motor and chopper drive are then powered on. In the chopper drive, the user selects variable wind mode to fetch real-time wind data. When the VEE Pro simulation is set to run mode, the chopper drive fetches real-time IoT-based wind data, and the DC motor starts rotating. The DFIG is then energized, and initially, the DC link voltage is set in the FPGA according to the parameters listed in Table 1. Additionally, the pulse switches (SW1–SW4) of the FPGA are activated to generate pulse signals for the RSC and GSC. During the investigation, the load remains steady, and key parameters such as torque, speed, tip-speed ratio, power factor, and real and reactive power are recorded. Finally, the load is reduced, and the chopper drive is de-energized to stop the system.

## Results and discussion

**Fig. 10:**
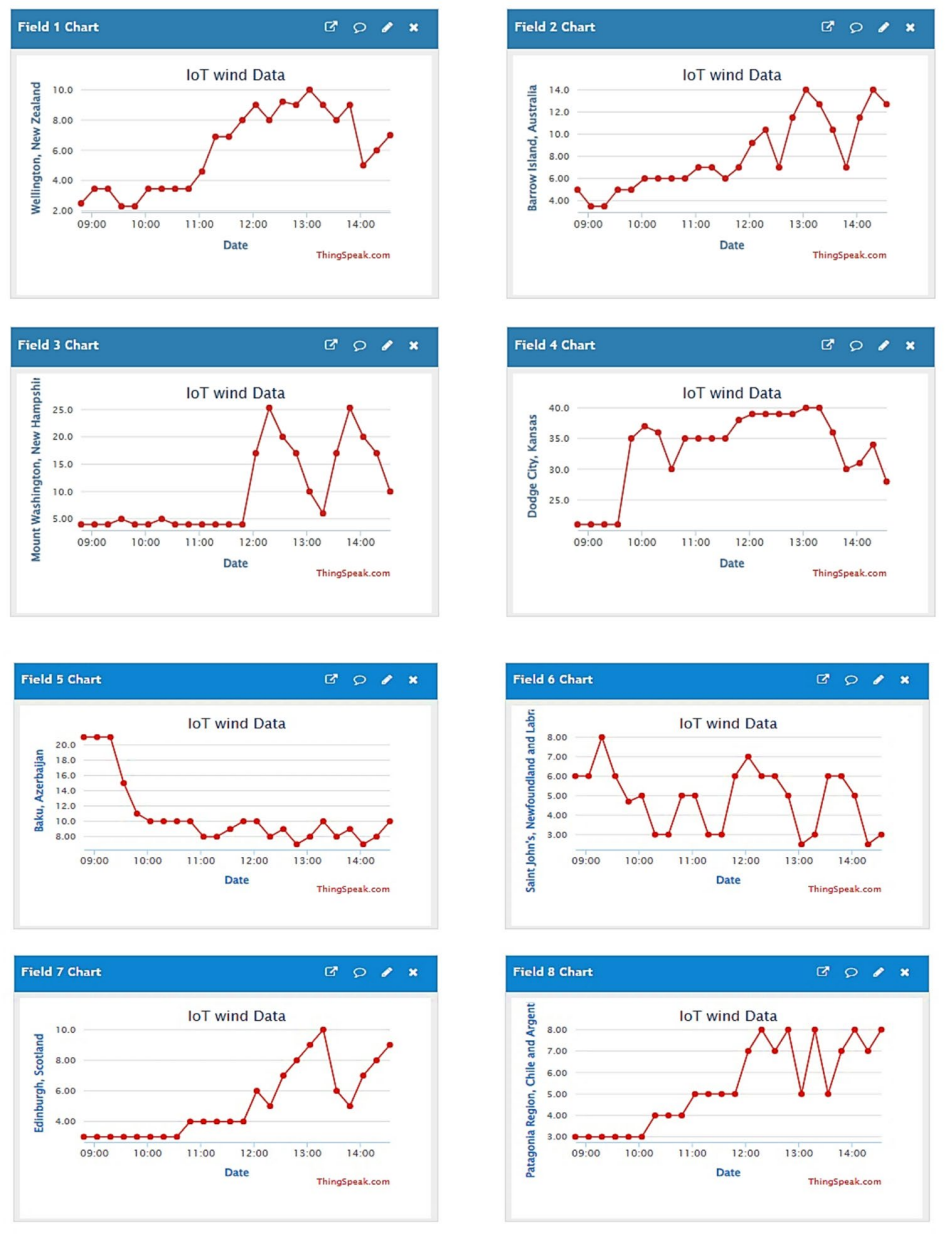
Real-time global wind speed data acquired through IoT-based cloud integration for wind emulator input.

To demonstrate the effectiveness of the proposed concept, extensive experimental analysis has been conducted. The main aim of this analysis is to validate the system’s accuracy in retrieving global wind scenarios through IoT and to evaluate the performance of the WPGS under those conditions. In the study, real-time wind data is collected via IoT from various locations worldwide. Users can utilize the interactive web interface to input latitude and longitude in Node-RED and access wind data for those locations from the OpenWeather API. In this research, locations such as Wellington, New Zealand; Barrow Island, Australia; Mount Washington, New Hampshire; Dodge City, Kansas; Baku, Azerbaijan; Saint John’s, Newfoundland; Edinburgh, Scotland; and the Patagonia Region, Chile, have been selected to gather wind scenario data. Additionally, an anemometer installed at the host institute feeds wind data into Node-RED. Figure 10 illustrates the real-time wind data retrieved through the IoT Cloud API from various global sites.

In this study, we examined the wind profile and scenarios of Barrow Island, Australia, as the wind profile illustrates the variation of wind speeds from 3 to 14 m/s. This wind profile data is then input into VEE Pro in CSV format; VEE Pro recreates the wind conditions into a digital signal to control the chopper-fed DC motor.

### Performance evaluation under fixed wind speed conditions

**Fig. 11:**
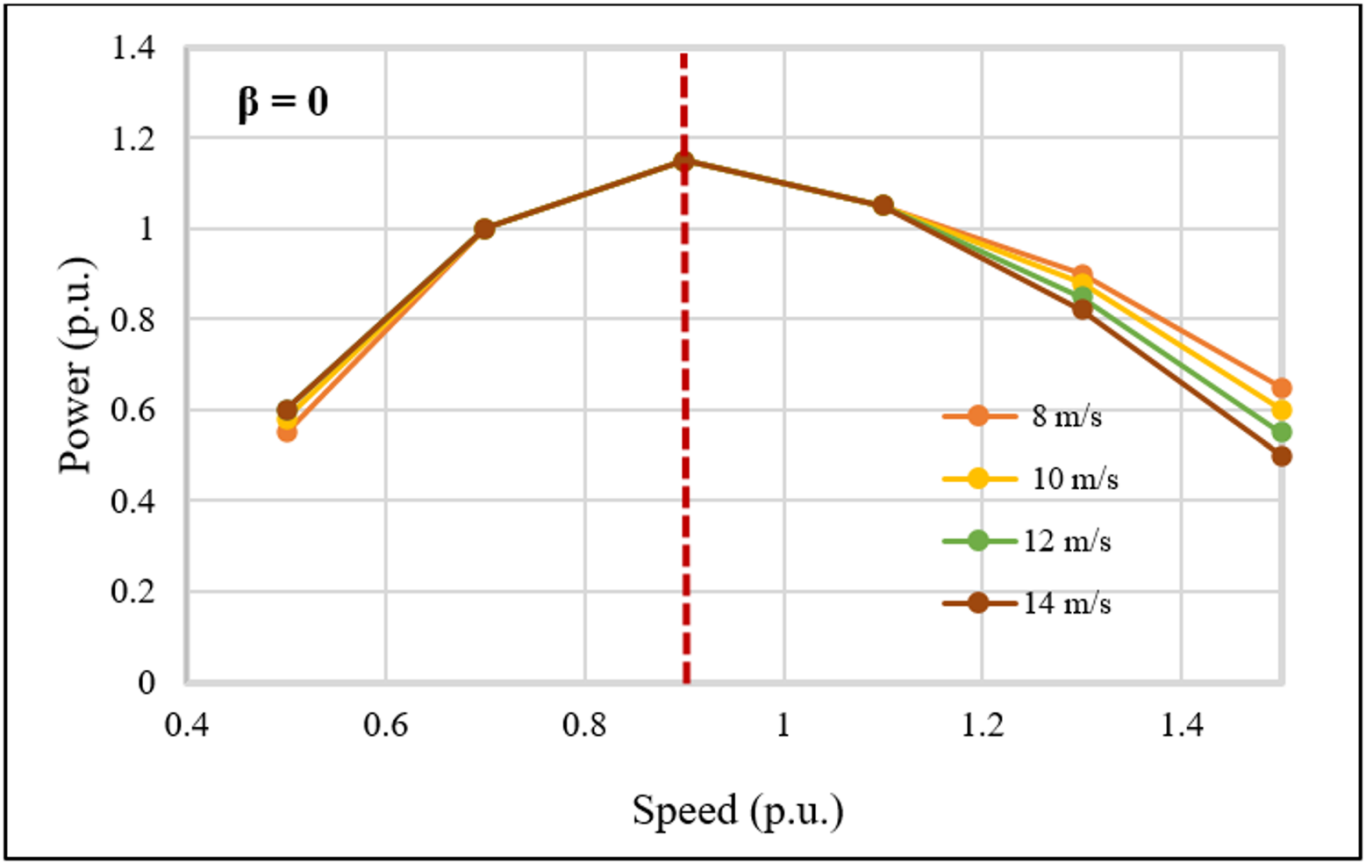
Power versus speed curve for fixed wind speed conditions.

The wind emulator system was evaluated under fixed wind speed conditions to assess the performance of the DFIG-WPGS. During the investigation, rotor speed, torque, and electrical power were observed at fixed wind speeds ranging from 8 to 14 m/s, with a pitch angle of β = 0°. Figure 11 displays the characteristic curve of the WPGS under these conditions. The power reaches its peak at λ = 9, demonstrating successful MPPT tracking amid varying aerodynamic conditions. This confirms the wind turbine’s emulated behavior as predicted by the theoretical Cp(λ,β) characteristics.

### Performance evaluation under variable wind speed conditions

**Fig. 12:**
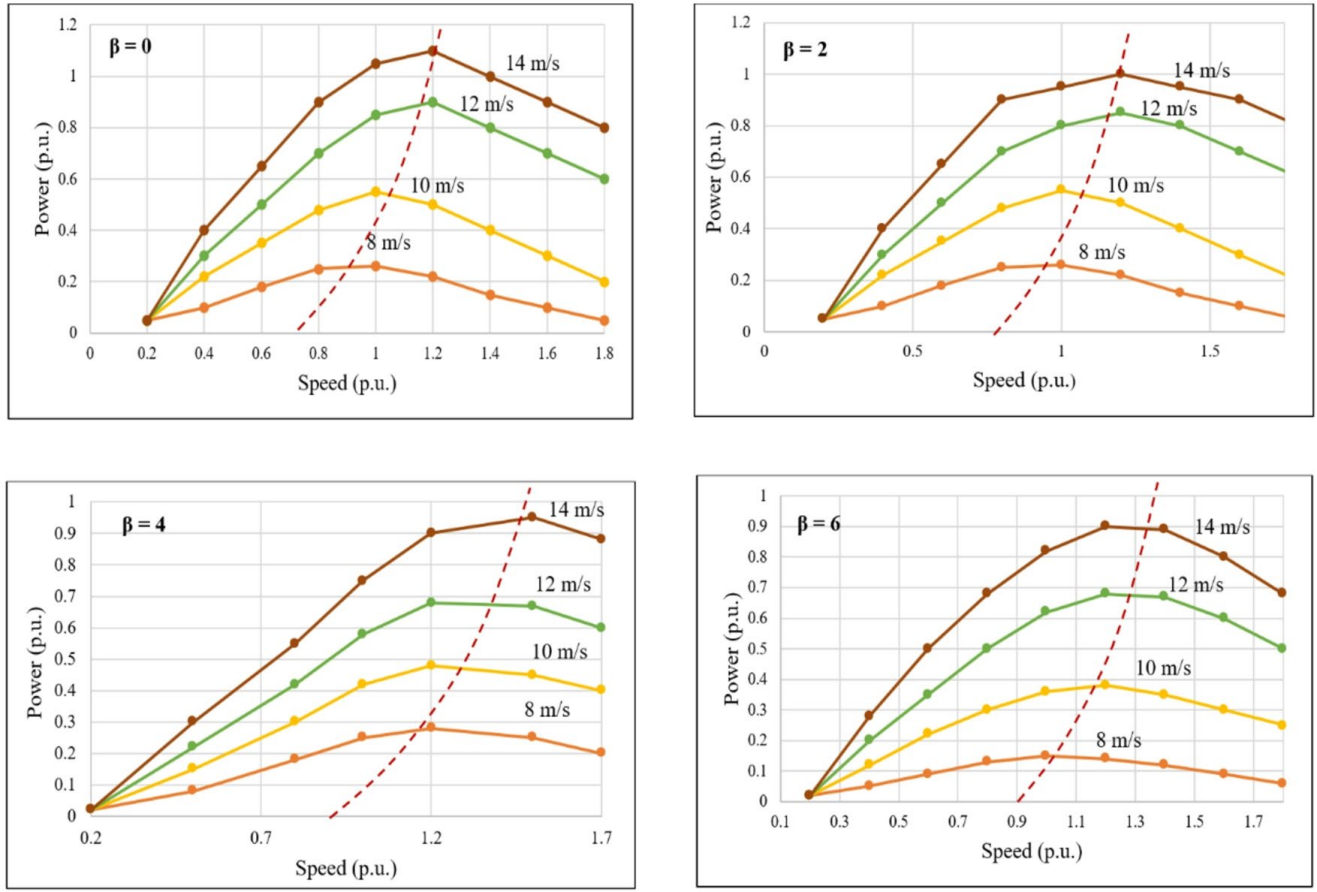
Power versus speed curve for variable wind speed conditions.

**Fig. 13:**
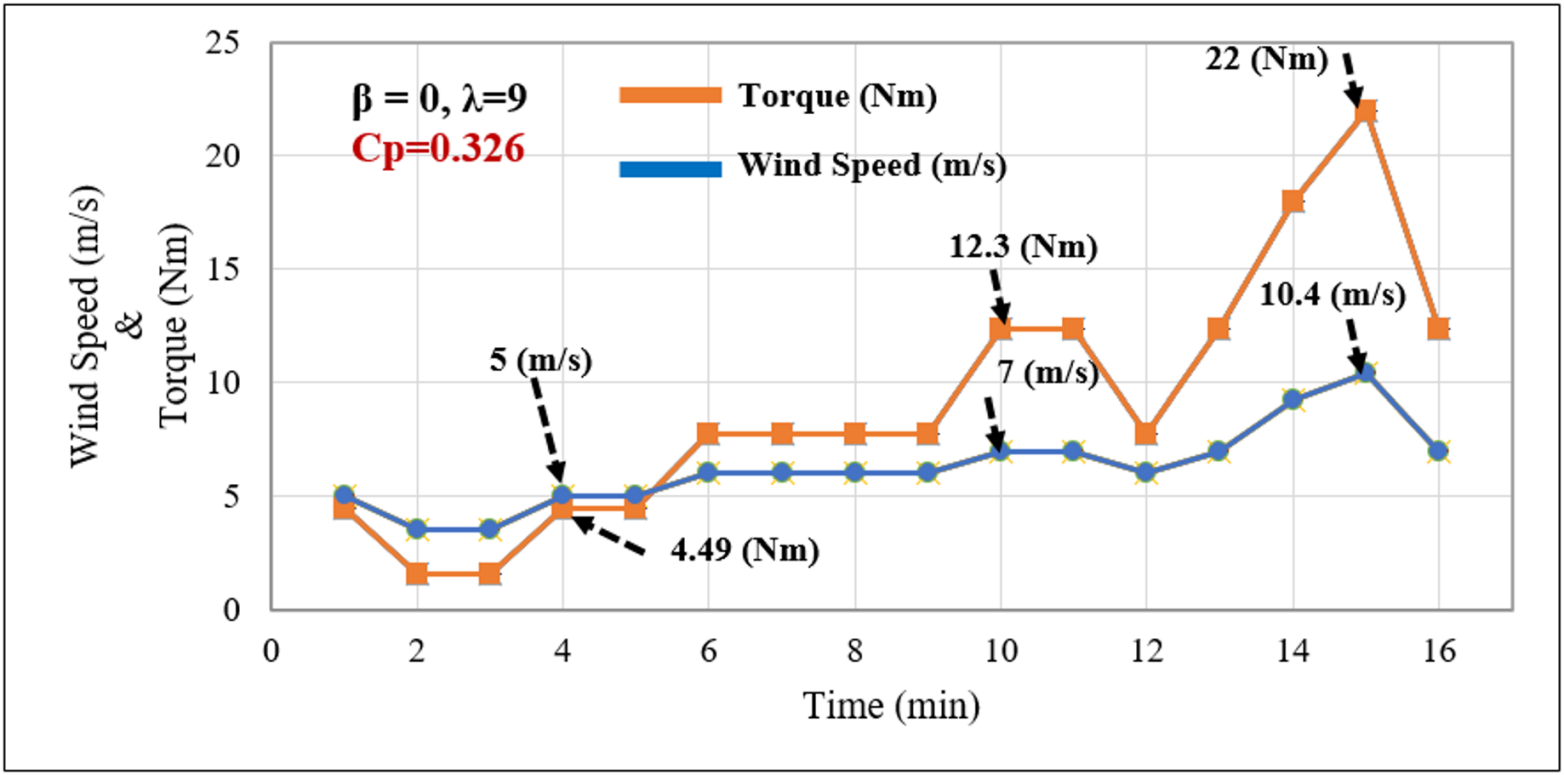
Wind speed versus torque curve.

Under variable wind speed conditions, the changing wind speeds are fed into the wind emulator. As wind speed varies, the rotor speed and generated torque change accordingly. Figure 12 shows the power-speed curve behavior for these conditions, where the torque responds precisely to wind speed fluctuations shown in Fig. 13. The chopper-fed DC drive ensures that the torque from the chopper is derived from Cp(λ,β), maintaining effectiveness in wind emulation. Additionally, any significant change is detected, and the corresponding torque is maintained in the system. It is also observed that as wind speed increases, rotor speed rises, and torque varies, reflecting the mechanical power produced by the emulated wind. The pitch angle significantly influences both speed and torque, aligning with the function of pitch control to regulate power capture.

**Table 4:** Experimental results for the DFIG-based wind emulation system.

Wind speed (m/s)	Pitch angle (β)	Speed (RPM)	Torque (Nm)	Grid parameters				Stator parameters			
				V (V)	I (A)	PF	P (W)	V (V)	I (A)	PF	P (W)
8	0	1232	15.5	405.72	1.8	0.999	718	325.38	2.5	-0.999	-800
	2	1160	16.46	405.36	1.7	0.999	673	325.38	3.0	-0.966	-936
	4	1100	17.36	406.08	1.6	0.998	644	325.96	2.6	-0.972	-812.8
9	0	1384	13.8	408.24	2.1	0.998	873	329.73	2.5	-0.99	-825.6
	2	1370	13.94	408.96	1.9	0.995	774	325.67	2.6	-0.961	-819.2
	4	1275	14.98	408.6	1.7	0.997	679	326.83	2.6	-0.991	-827.2
10	0	1530	12.48	407.16	2.4	0.999	995	328.28	2.6	-0.987	-830.4
	2	1515	12.61	410.4	2.3	0.998	924	328.57	2.6	-0.969	-827.2
	4	1510	12.65	407.88	2.2	0.993	893	330.02	2.5	-0.981	-819.2
11	0	1487	12.84	400.68	2.3	0.995	923	322.77	2.6	-0.975	-824
	2	1436	13.3	406.8	2.2	0.99	884	329.15	2.6	-0.967	-819.2
	4	1412	13.53	407.52	2.0	0.998	829	328.28	2.6	-0.973	-835.2
12	0	1425	13.4	403.92	2.1	0.981	848	328.28	2.5	-0.985	-819.2
	2	1353	14.12	408.96	1.9	0.997	773	329.44	2.6	-0.971	-836.8
	4	1332	14.34	408.96	1.8	0.997	744	329.44	2.6	-0.971	-836.8

Table 4 presents the experimental results for the DFIG-based wind emulation system under different wind speeds and pitch angles. The tests cover wind speeds from 8 m/s to 12 m/s, with pitch angles of 0, 2, and 4 degrees. This highlights the system’s flexibility in assessing WPGS performance across various operational scenarios. As shown in Table 2, rotor speed rises with increasing wind velocity but falls as the pitch angle increases. For example, at 8 m/s and β = 0°, the speed was 1232 RPM, whereas it decreased to 1100 RPM for β = 4°. This is due to a reduction in aerodynamic efficiency as β increases, which limits the effective rotor swept area. Additionally, torque slightly increased with higher β because of slower rotor speeds, as torque is inversely related to speed for a constant mechanical power (Eq. 4). The highest torque of 17.36 Nm was recorded at 8 m/s and β = 4°, while a lower torque of 12.48 Nm was observed at 10 m/s and β = 0°.

Furthermore, the actual power measured at the stator and grid terminals of the DFIG is shown in Table 2. It can be seen that, at 10 m/s and β = 0°, the active power supplied to the grid reached 995 W, while reactive power stayed close to zero, confirming a near-unity power factor (*pf* ≈ 0.999), which indicates proper control of the GSC and RSC converters. Voltage levels were effectively regulated by the FPGA-based controller, as demonstrated by the grid and stator voltages ranging from 322 V to 410 V. Regarding the grid parameters, the grid voltage remains stable around 400 V, and the power factor stays close to 1 (around 0.9), showing efficient power transfer to the grid. The real power fed into the grid increases with wind speed, confirming the DFIG-WPGS’s capability to deliver energy to the grid as wind conditions improve. For the stator parameters, the stator voltage remains stable between 320 and 330 V. Notably, the stator real power values are negative, indicating that the stator absorbs power from the grid under these operating conditions, which is typical for DFIGs, especially when providing reactive power compensation. The stator power factor values are also negative, indicating reactive power consumption.

**Fig. 14:**
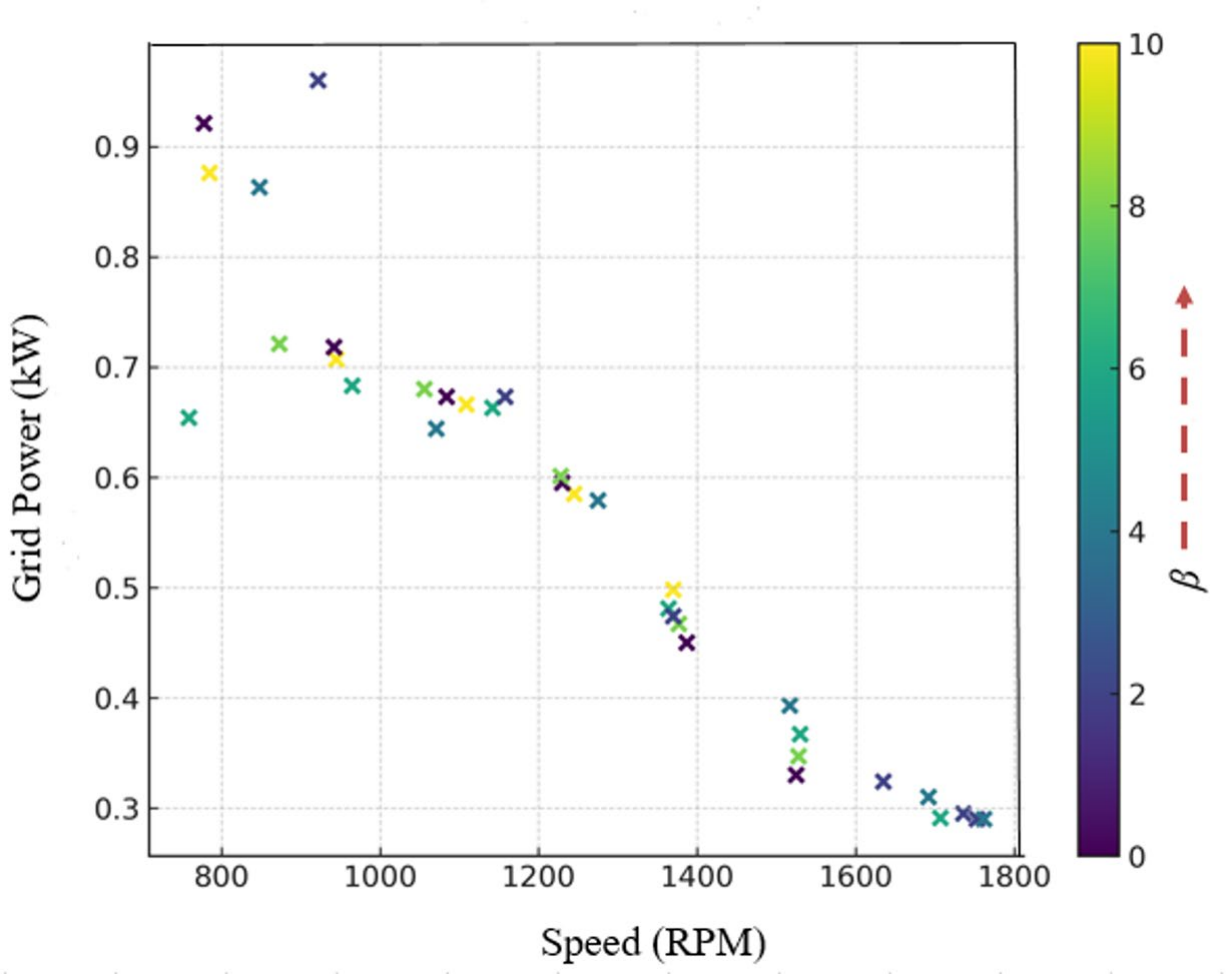
Validation of pitch angle control on grid power output in the wind emulator.

Figure 14 shows the scatter plot relating turbine rotor speed and grid power for various pitch angles. From the graph, it is clear that the wind turbine emulator performs optimal power tracking. At a lower pitch angle (i.e., β = 0°), the turbine produces moderate speed and optimal grid power. Additionally, under high wind speed conditions, the pitch angle increases, which reduces grid power and decreases the torque of the wind emulator to protect the system. This validation confirms that maximum power tracking occurs when implementing the proposed concept in the wind emulator system.

**Fig. 15:**
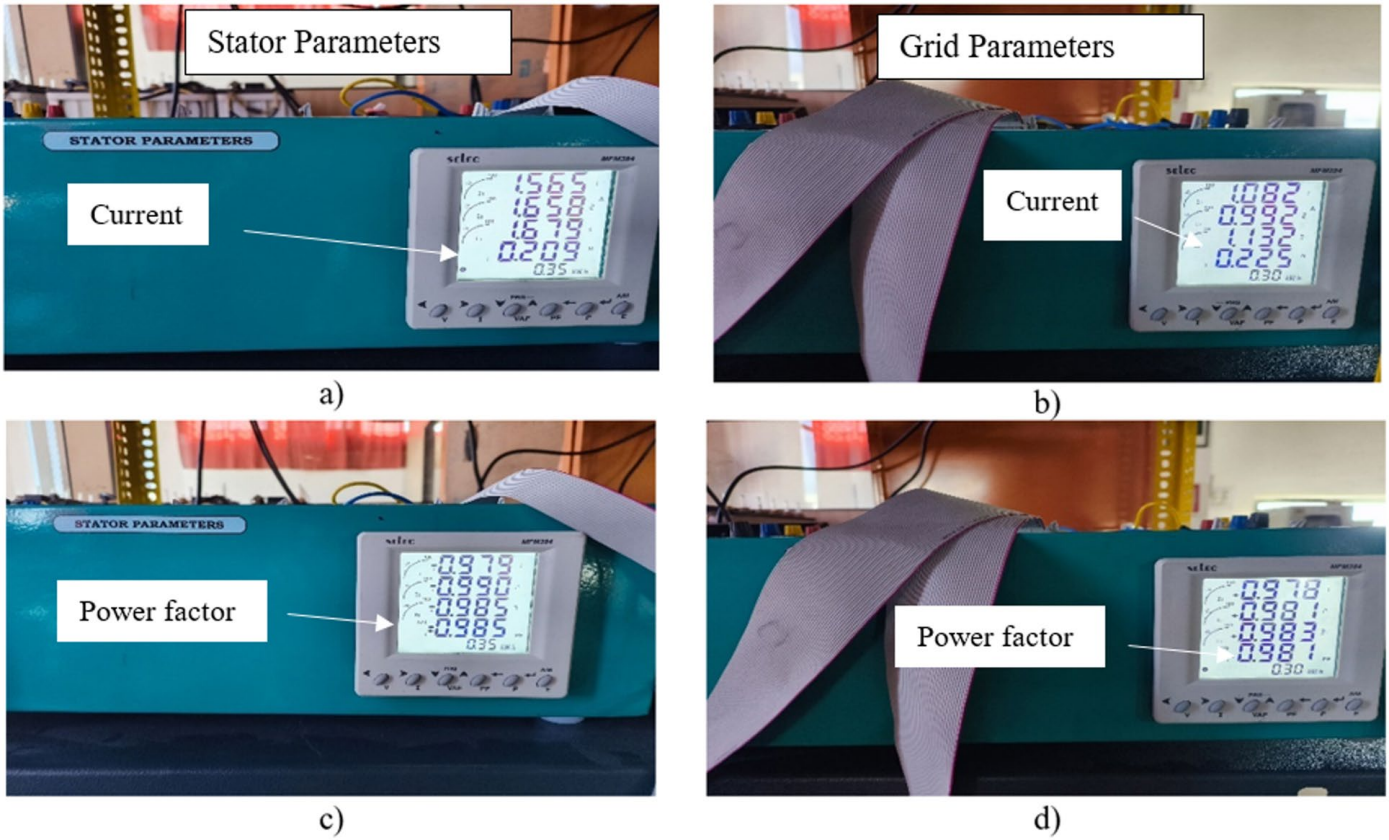
Measured stator and grid parameters.

Figure 15 shows the measured electrical parameters of both the stator and grid terminals of the DFIG-WPGS. The readings were taken using multifunction energy meters. During operation, the DC-link voltage of the back-to-back converter is set to 375 V to facilitate power flow control. Figure 15a and b display the approximate currents of the stator and grid. The stator current is around 2.5 A, which aligns with the expected value at rated operating conditions. Meanwhile, the grid current is about 1.8 A, indicating active power transfer from the generator to the grid through the regulated grid-side inverter. Figure 15c and d show the approximate power factors of the stator and grid. The stator power factor is measured at -0.985, indicating lagging reactive power under certain loading conditions. On the grid side, the power factor is approximately 0.999, reflecting reactive power compensation and power factor correction by the GSC to deliver high-quality power into the grid.

**Fig. 16:**
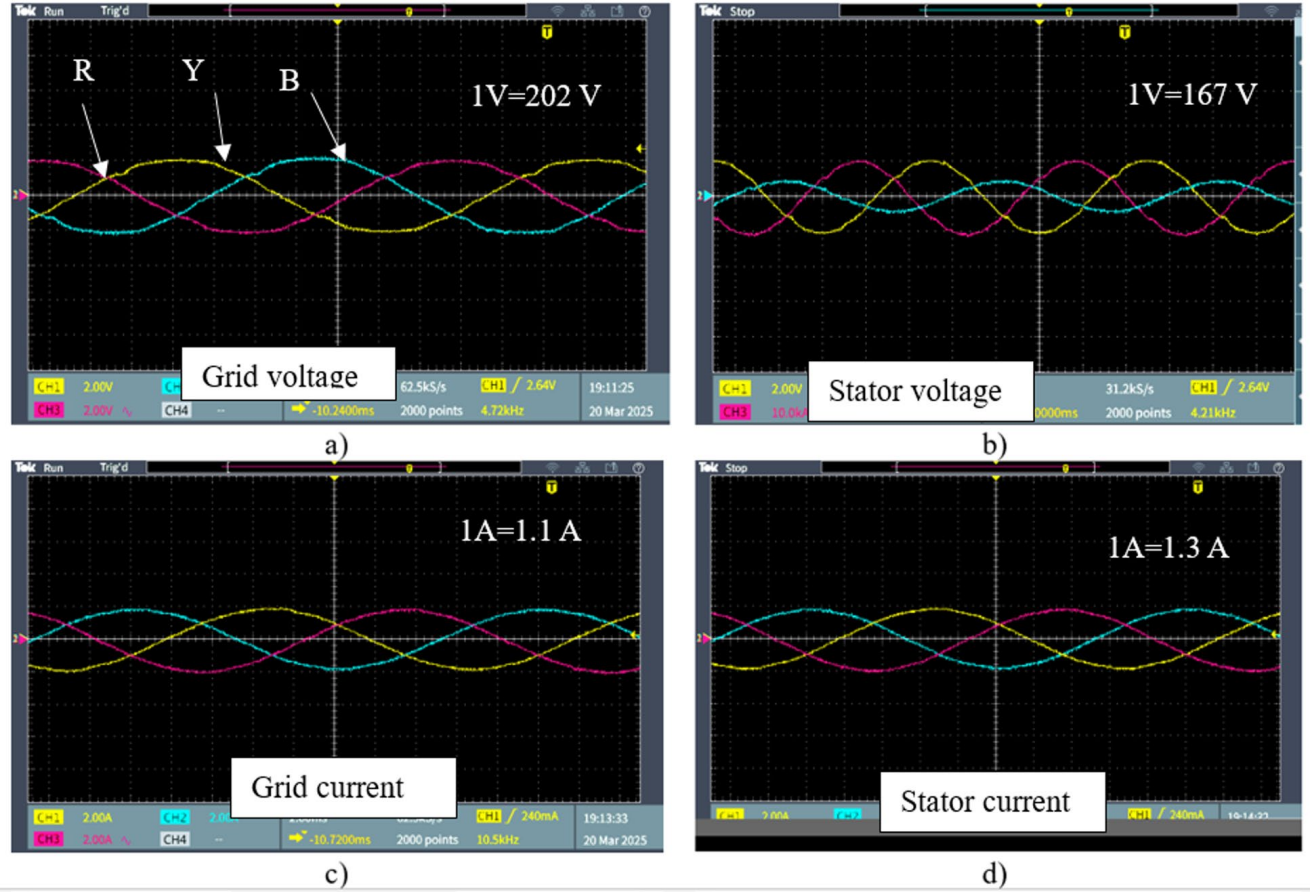
Real-time WPGS measurements: (**a**) grid voltage, (**b**) stator voltage, (**c**) grid current, and (**d**) stator current.

**Fig. 17:**
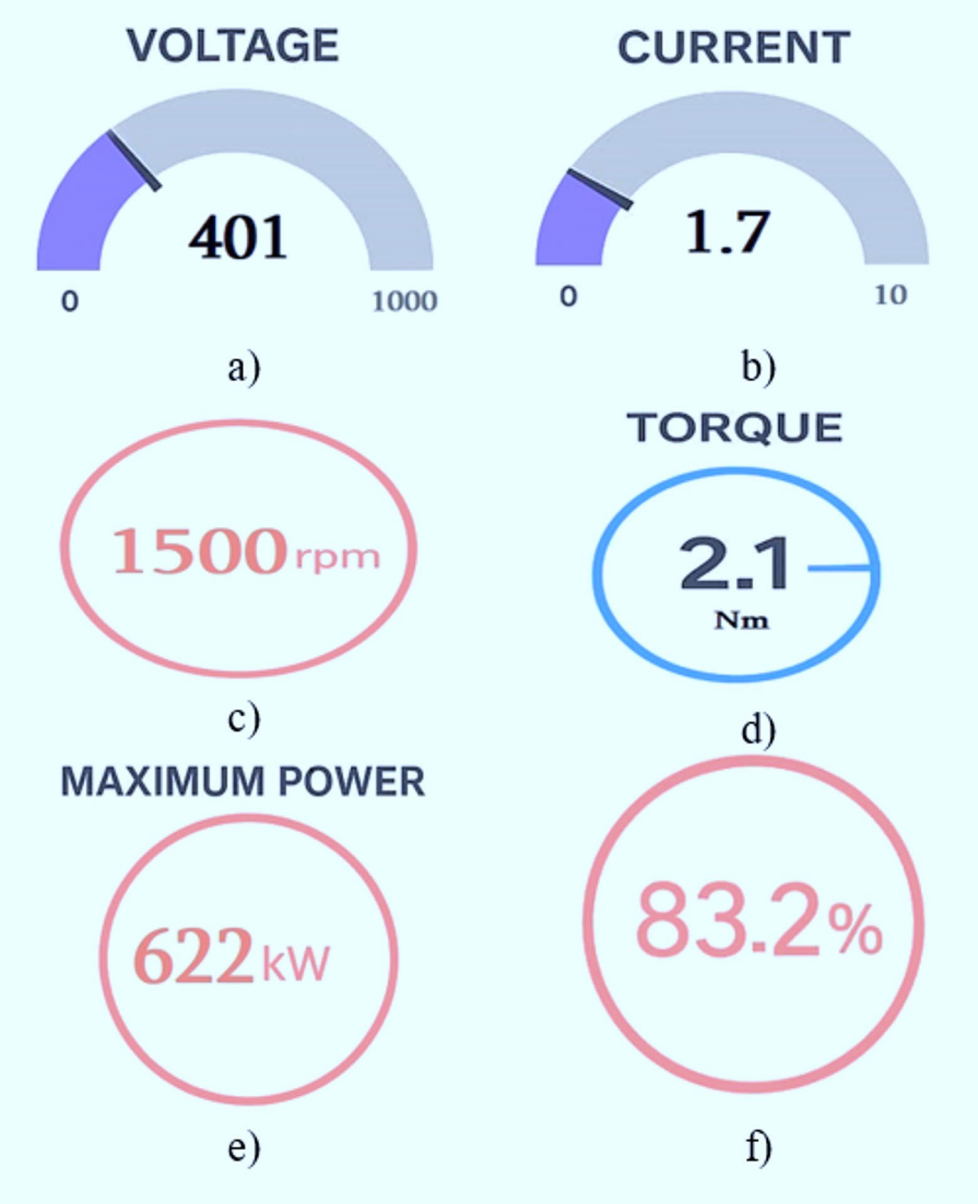
Wind emulator results in Node-RED dashboard: (**a**) voltage, (**b**) current, (**c**) speed, (**d**) torque, (**e**) power, and (**f**) efficiency

Figure 16 displays the output waveforms of the stator and grid-side voltage and current for the DFIG wind emulator system. Figure 16a and c illustrate the three-phase grid voltage and current, with measured RMS values of 412 V and 2.39 A, respectively. The output confirms proper synchronization with the grid. Figure 16b and d show the three-phase stator voltage and current, with RMS values of 304 V and 2.6 A. It is evident that the stator voltage waveform exhibits distortion due to the DFIG’s internal impedance and energy conversion losses. The waveform verifies that the stator voltage aligns with the expected phase sequence and supports stable power delivery. In this approach, the FPGA controller effectively manages the regulation of the stator and rotor side converters and maintains a DC-link voltage of 375 V for optimal power synchronization with the grid. Figure 17 presents the wind emulator results, including voltage, current, speed, torque, power, and efficiency, all visualized on the Node-RED dashboard. Furthermore, the accuracy for the proposed emulator is calculated by the Mean Absolute Percentage Error (MAPE) between theoretical aerodynamic power *P*_*m*_ and emulator-measured power *P*_*ex*_^30^.

18 $$MAPE=\frac{1}{n}\sum\limits_{{i=1}}^{n} {\left|\frac{{{P_m} - {P_{ex}}}}{{{P_m}}}\right|\times 100}$$

The calculated MAPE gave 87% accuracy with the correlation coefficient *r* = 0.964, indicating high fidelity between simulated and real-time performance curves.

### WPGS health index analysis

**Fig. 18:**
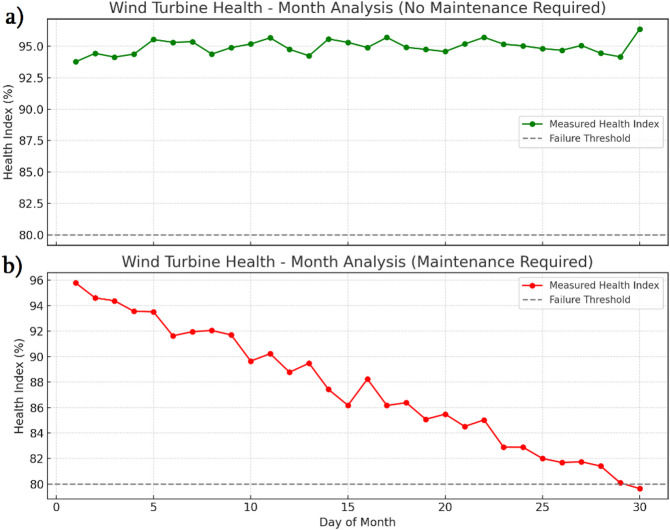
Wind turbine health index predictive analysis.

Predictive maintenance analysis is a data-driven method used to determine the best time to perform maintenance before a failure happens. In this approach, real-time condition monitoring data such as vibration, temperature, power output, and rotor speed are collected from IoT-enabled sensors and processed to evaluate the current health of the equipment. A Health Index (HI) is calculated by normalizing these condition parameters as,

19 $$HI=\frac{{\sum_{{i=1}}^{n} {{w_i}\left[ {1 - \frac{{{x_i}}}{{{x_{i,\text{max} }}}}} \right]} }}{{\sum_{{i=1}}^{n} {{w_i}} }}\times 100$$

where *x*_*i*_ is the measured value of parameter *i*,* x*_*i*, max_ is the failure threshold for parameter *i*,* and w*_*i*_ represents the weight for parameter *i.* By plotting the HI over time, a degradation trend is identified using machine learning-based regression analysis. Figure 18 shows the HI of the studied wind turbine system. In the investigation, the failure threshold is set to 80%. Figure 18a shows the healthy condition of the system, for which the investigation predicts that maintenance is not required. In further analysis, the system is subjected to external vibration via an auxiliary motor to demonstrate the predictive maintenance capability. As shown in Fig. 18b, the HI% gradually decreases over time, and the analysis forecasts that maintenance will be necessary. This predictive monitoring framework ensures that the wind turbine operates efficiently, enabling maintenance interventions to be performed only when needed, thereby preventing unexpected failures and optimizing operational costs.

## Conclusion

This study explores a new, real-time IoT-enabled wind emulator system for testing the performance of DFIG-based WPGS under different wind speed conditions. It collects wind speed data globally via IoT and cloud services and processes this data with the emulator to assess the WPGS performance. The setup includes a 1 kW Doubly-Fed Induction Generator (DFIG) connected to a Brushless DC (BLDC) motor, with the wind turbine modeled on the VEE Pro platform that integrates an IoT cloud API and FPGA controller to simulate real-world wind scenarios. The key findings are as follows,

- The IoT-based wind emulator system effectively reproduces real-time wind speeds measured between 3 and 19 m/s using IoT and cloud services.
- Optimal power tracking occurs at β = 0°, with the turbine reaching a maximum power coefficient of Cp = 0.326 and an optimal tip speed ratio of λ = 9 to generate maximum grid power. Meanwhile, when wind speed exceeds the cut-out speed, β increases, which lowers turbine torque and grid power.
- The negative value measured in the stator power and power factor confirms reactive power compensation, which is typical for DFIG operational characteristics.
- A unity power factor of 0.999 is measured on the grid side, indicating that the FPGA controller effectively manages the stator and grid converter to supply high-quality power. This demonstrates the performance of the DFIG-WPGS.
- WPGS health index monitoring offers predictive maintenance needs, which decrease downtime and greatly enhance grid integration reliability.

The results confirm the accuracy, flexibility, and practical use of the proposed emulator system for wind turbine testing and grid integration studies. The future scope of the work can be expanded by integrating AI-based analytics. Additionally, further studies on grid stability, hybrid emulation, and enhanced cybersecurity will improve their practical relevance and scalability.

## Data Availability

The data used and/or analyzed during the current study are available from the corresponding author on reasonable request.
